# mRNA Vaccine Mitigates SARS-CoV-2 Infections and COVID-19

**DOI:** 10.1128/spectrum.04240-22

**Published:** 2023-01-25

**Authors:** Christina M. Kackos, Sherri L. Surman, Bart G. Jones, Robert E. Sealy, Trushar Jeevan, Christopher J. H. Davitt, Sergei Pustylnikov, Tamarand L. Darling, Adrianus C. M. Boon, Julia L. Hurwitz, Marcelo M. Samsa, Richard J. Webby

**Affiliations:** a Department of Infectious Diseases, St. Jude Children’s Research Hospital, Memphis, Tennessee, USA; b St. Jude Children’s Research Hospital Graduate School of Biomedical Sciences, Memphis, Tennessee, USA; c GreenLight Biosciences, Woburn, Massachusetts, USA; d Department of Medicine, Washington University School of Medicine, St. Louis, Missouri, USA; e Molecular Microbiology, Washington University School of Medicine, St. Louis, Missouri, USA; f Pathology and Immunology, Washington University School of Medicine, St. Louis, Missouri, USA; Johns Hopkins Hospital

**Keywords:** SARS-CoV-2, coronavirus, mRNA, vaccines, virology

## Abstract

The novel coronavirus, severe acute respiratory syndrome coronavirus 2 (SARS-CoV-2), was identified in December of 2019 and is responsible for millions of infections and deaths across the globe. Vaccination against SARS-CoV-2 has proven effective to contain the spread of the virus and reduce disease. The production and distribution of these vaccines occurred at a remarkable pace, largely through the employment of the novel mRNA platform. However, interruptions in supply chain and high demand for clinical grade reagents have impeded the manufacture and distribution of mRNA vaccines at a time when accelerated vaccine deployment is crucial. Furthermore, the emergence of SARS-CoV-2 variants across the globe continues to threaten the efficacy of vaccines encoding the ancestral virus spike protein. Here, we report results from preclinical studies on mRNA vaccines developed using a proprietary mRNA production process developed by GreenLight Biosciences. Two mRNA vaccines encoding the full-length, nonstabilized SARS-CoV-2 spike protein, GLB-COV2-042 and GLB-COV2-043, containing uridine and pseudouridine, respectively, were evaluated in rodents for their immunogenicity and protection from SARS-CoV-2 challenge with the ancestral strain and the Alpha (B.1.1.7) and Beta (B.1.351) variants. In mice and hamsters, both vaccines induced robust spike-specific binding and neutralizing antibodies, and in mice, vaccines induced significant T cell responses with a clear Th1 bias. In hamsters, both vaccines conferred significant protection following challenge with SARS-CoV-2 as assessed by weight loss, viral load, and virus replication in the lungs and nasopharynx. These results support the development of GLB-COV2-042 and GLB-COV2-043 for clinical use.

**IMPORTANCE** SARS-CoV-2 continues to disrupt everyday life and cause excess morbidity and mortality worldwide. Vaccination has been key to quelling the impact of this respiratory pathogen, and mRNA vaccines have led the charge on this front. However, the emergence of SARS-CoV-2 variants has sparked fears regarding vaccine efficacy. Furthermore, SARS-CoV-2 vaccines continue to be unevenly distributed across the globe. For these reasons and despite the success of emergency authorized and licensed SARS-CoV-2 vaccines, additional vaccines are needed to meet public health demands. The studies presented here are significant as they demonstrate robust protective efficacy of mRNA vaccines developed by GreenLight Biosciences against not only wild-type SARS-CoV-2, but also Alpha and Beta variants. These results support the progression of GreenLight Biosciences SARS-CoV-2 mRNA vaccines to clinical trials as another defense against SARS-CoV-2.

## INTRODUCTION

In December of 2019, severe acute respiratory syndrome coronavirus 2 (SARS-CoV-2) emerged in Wuhan, China, and quickly spread to the rest of the globe, prompting the World Health Organization (WHO) to declare SARS-CoV-2 a pandemic virus by March 2020. At the time of writing, SARS-CoV-2 has caused over 601 million infections and over 6.4 million deaths worldwide (1, 2). Due to limited treatment options for coronavirus disease 2019 (COVID-19), physical measures have been employed, including social distancing and the use of facial masks. However, to effectively quell the SARS-CoV-2 pandemic, vaccines are urgently needed. This has prompted a surge of research into SARS-CoV-2 vaccine development and testing, resulting in several candidate vaccines receiving FDA Emergency Use Authorization and, in some cases, licensure (3–5).

Two of these vaccines, produced by BioNTech/Pfizer and Moderna, use mRNA transcripts encoding the ancestral SARS-CoV-2 spike protein with a 2 proline prefusion stabilization (S-2P); these vaccines have been shown to be >90% effective in preventing symptomatic COVID-19 caused by the ancestral strain of the virus (6, 7). However, challenges in manufacture due to reagent shortages and supply chain disruptions coupled with the need to vaccinate billions of people have resulted in a vaccine supply that has struggled to meet global demand (8). Furthermore, this slow global distribution continues to provide SARS-CoV-2 a window within which to evolve, resulting in the emergence of variant viruses. While early studies assessing the efficacy of the BioNTech/Pfizer vaccine, COMIRNATY, estimated ~75% efficacy against Beta infection in a cohort in Qatar, clinical and real-world cases in Israel and the United States showed a vaccine effectiveness of ~55% against the Beta variant (9). A California cohort assessing the efficacy of the Moderna vaccine, Spikevax, found a vaccine effectiveness of 98.4% against Alpha infection in individuals who received two doses of the vaccine though this effectiveness declined over time (10).

mRNA vaccines can be designed and produced rapidly, requiring only the nucleotide sequence of the target antigen, and can be customized to enhance immunogenicity and stability (11, 12). Animal studies have demonstrated the ability of mRNA vaccines to protect from a variety of pathogens, including influenza virus (13, 14), Zika virus (15, 16), rabies virus (17, 18), and HIV-1 (19), and the successes of the BioNTech/Pfizer and Moderna vaccines have begun to answer the outstanding questions of vaccine safety and efficacy in humans. Though the manufacture of these vaccines is reasonably scalable, high-yield production remains a hurdle as some reagents are not readily available in the appropriate grade for clinical use (12).

Here, we present data on mRNA vaccines produced using Greenlight Biosciences’s production process. Two constructs, GLB-COV2-042 (−042) and GLB-COV2-043 (−043), containing unmodified and modified nucleotides, respectively, encoding the SARS-CoV-2 full-length ancestral spike protein and encased in lipid nanoparticle (LNP) carriers were assessed. Both vaccines elicited potent neutralizing spike-specific antibody responses, induced spike-specific T cell responses with a Th1-bias, and conferred significant protection against the ancestral, Alpha, and Beta strains of SARS-CoV-2 in the hamster model, reducing both morbidity and viral replication in the lungs and nasopharynx. The results encourage advancement of GLB-COV2 mRNA vaccines to clinical trials.

## RESULTS

### *In vitro* expression of GLB-COV2 mRNA.

Spike protein expression was analyzed by Western blot following mRNA transfection in 293 T cells. −042 and −043 produced by GreenLight Biosciences and with a commercially available IVT kit were transfected; recombinant S1 protein was used as a positive control. The full-length spike protein is detected at a molecular weight of ~180 kDa with some variations in size due to glycosylation. At 48-h posttransfection, bands of the appropriate size could be visualized in all −042 and −043 lanes with no obvious differences in expression between GLB-RNA and IVT-RNA, indicating GLB-COV2 mRNA vaccines resulted in expression of the full-length spike protein (Fig. S1a).

Spike protein expression was further analyzed by enzyme-linked immunosorbent assay (ELISA). Again, GLB-RNA and IVT-RNA −042 and −043 mRNAs were transfected in 293 T cells; three negative controls were included in the experiment: mock-transfected cells, a firefly luciferase mRNA, and an EGFP mRNA. Protein expression was evaluated 24-h posttransfection. GLB-COV2 mRNA vaccines scored significantly above assay background as determined by the negative controls, indicating positive spike protein expression (Fig. S1b). Overall, −042 and −043, exhibited expression of the SARS-CoV-2 full-length spike protein with very similar potency regardless of whether they were generated by commercially available IVT kit or GLB RNA synthesis.

### Vaccine immunogenicity in mice. (i) SARS-CoV-2-specific antibodies.

**FIG 1:**
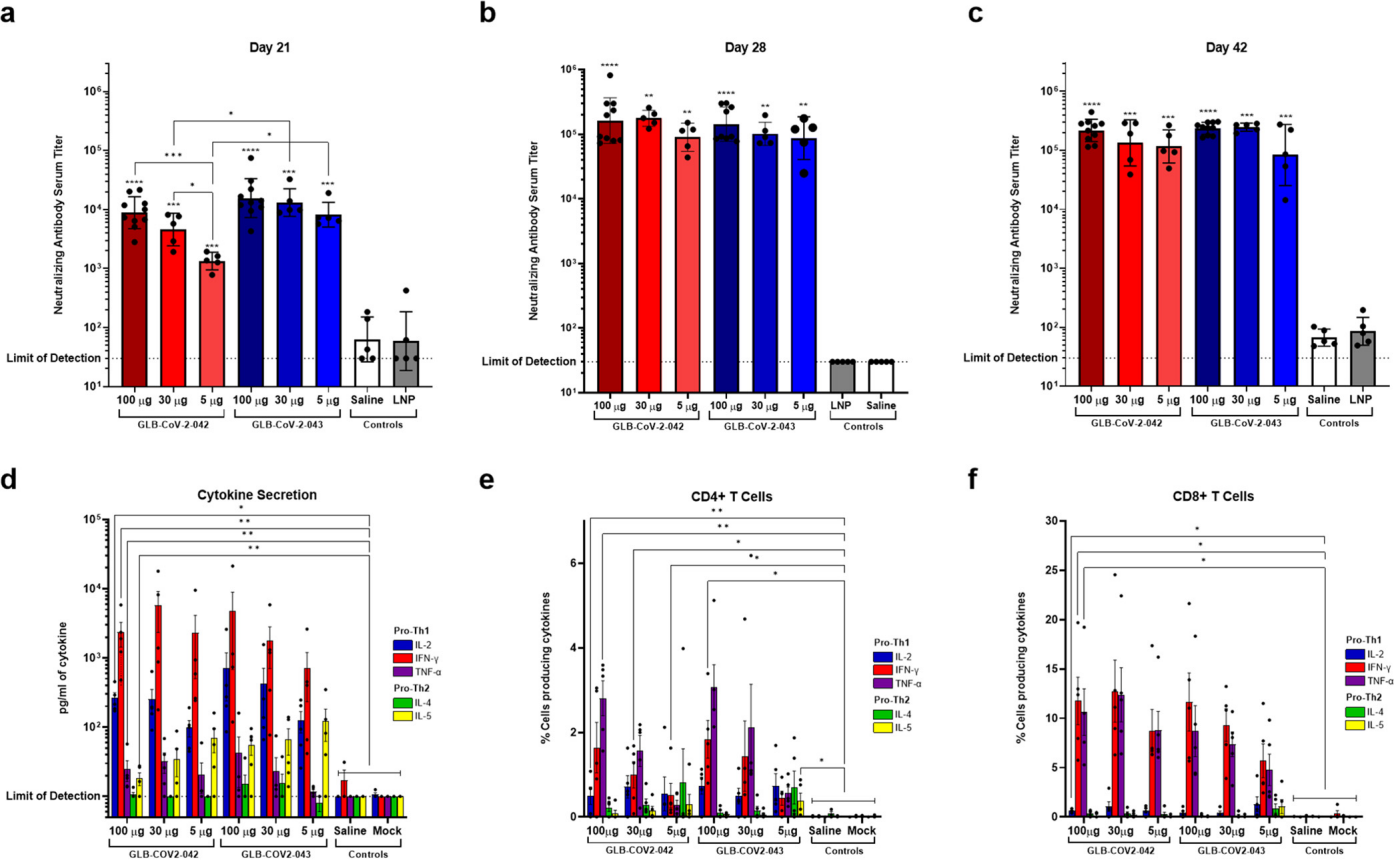
Immunogenicity in mice following GLB-SARS-CoV mRNA vaccination. 8-week-old female C57BL/6 mice were immunized with 100 μg (*n* = 5), 30 μg (*n* = 5), or 5 μg (*n* = 5) of GLB-COV2-042 or GLB-COV2-043 mRNA on days 0 and 21. Blood was collected on days 0, 28, and 42 for serum antibody analysis. Spleens for T cell analysis were collected ~3 months postinitial immunization. (a) Postprime neutralizing antibody titers on day 21 (b) Postboost neutralizing antibody titers on day 28. (c) Postboost neutralizing antibody titers on day 42. (d) Cytokine secretion of restimulated splenocytes. (e) Percent of CD4^+^ T cells secreting Th1/2 cytokines. (f) Percent of CD8^+^ T cells secreting Th1/2 cytokines. Means and standard deviations are shown. Data from controls were combined for statistical analyses. Statistical analyses were performed using rank-based Mann-Whitney and Holm-Šidάk multiple-comparison tests. Statistical signifiers above bars represent significance between that group and controls. *, *P* < 0.05; ***, *P* < 0.005; ****, *P* < 0.001.

The immunogenicity of −042 and −043 mRNA constructs was first assessed by immunizing 8-week-old female C57BL/6 mice on days 0 and 21 with vaccine doses of 100 μg, 30 μg, or 5 μg. These doses were chosen to be consistent with the Spikevax and COMIRNATY adult dosing guidelines of two administrations of 100 and 30 μg, respectively, as well as evaluate the effectiveness of GLB-COV2 mRNA vaccines below these concentrations (6, 7). The neutralizing activity of vaccine induced antibodies against the homologous virus SARS-CoV-2 USA-WA1/2020 (WA-1) was determined using a focus reduction neutralization test (FRNT) at days 21, 28, and 42 postinitial immunization (Fig. 1a to c). On day 21, no difference in neutralizing antibody (Nab) titers were detected between −042 and −043 at the 100 μg dose (geometric mean titers [GMT] of 8,852.35 and 15,574.5, respectively, *P* = 0.08921); however, Nab titers following immunization with 30 μg and 5 μg of −043 (GMTs of 13,130.2 and 8,187.63, respectively) were significantly higher compared to the corresponding doses of −042 (GMTs of 4,575.74 and 1,337.93, respectively, *p* < 0.05) (Fig. 1a). At this time −042 resulted in induction of significantly higher Nab titers at the 100 μg dose compared to the 30 and 5 μg doses, while mice that received −043 exhibited no dose effects (Fig. 1a). One-week postboost (day 28), both GLB-COV2 mRNA vaccines induced high neutralizing antibody titers (GMTs of 152592.2, 177241, and 89796.03 for 100, 30, and 5 μg of −042, respectively, and 143789.4, 101657, and 87065.93 for 100, 30, and 5 μg of −043) at all doses compared to control animals with no difference in Nab titer between vaccine or doses detected (Fig. 1b). At 3-weeks postboost (days 42), GLB-COV2 mRNA vaccine Nab titers remained high (GMTs of 217,653, 134,073, and 116,439 for 100, 30, and 5 μg of −042, respectively, and 236,012, 246,715, and 83,448.9 for 100, 30, and 5 μg of −043) at all doses compared to control animals. At this time point, Nab titers in the 30 and 5 μg groups of −042 and −043 rose from day 28, increasing the statistical significance of these groups from *P* < 0.01 to *P* < 0.0001 compared to control groups (Fig. 1c). No difference in Nab titer between vaccines or doses were detected (Fig. 1c).

**(ii) T cell stimulation and cytokine production.** For analyses of virus-specific T cell responses, immunized and naive age-matched control C57BL/6 mice were sacrificed ~2 months after the second vaccination for spleen collection. Two different cytokine analyses were performed. One assay evaluated cytokine production in the supernatant after stimulation of 1× 10^6^ T cells *in vitro* overnight. Cells were stimulated with a pool of 253 unique 15-mer peptides, overlapping by 10 amino acids and covering the entire SARS-CoV-2 spike protein. Bead based multiplex assays were used to measure the concentration of pro-Th1 and pro-Th2 cytokines in the supernatant 18 h after peptide restimulation. In addition to assays of cell supernatants, intracellular cytokine (ICC) assays were used to measure cytokine production in CD4^+^ and CD8^+^ T cell populations taken 5 h to 6 h after peptide restimulation. Biases toward Th1 or Th2 cytokine profiles were examined (20). Secreted cytokines, including IL-2, IFN-γ, TNF-α, IL-4, and IL-5, are shown in Fig. 1d. In all cases, peptide-specific responses were robust, and cells displayed a preference for expression of Th1 cytokines, IL-2 and IFN-γ, compared to Th2 cytokines, IL-4 and IL-5.

For ICC assays, cells were stained for surface markers CD3 (BV711-labeled antibodies), CD4 (APC-labeled antibodies), and CD8 (APC Cy7-labeled antibodies) and for the presence of intracellular cytokines with antibodies against IFN-γ (FITC-labeled antibodies), TNF-α (PE Dazzle 594-labeled antibodies), IL-2 (PE-labeled antibodies), IL-4 (BV605-labled antibodies), and IL-5 (BV421-labeled antibodies). Lymphoid cells were gated, after which the percentages of cytokine-positive cells were determined among CD4^+^ T cells (CD3+CD4^+^) and CD8^+^ T cells (CD3+CD8^+^; see Fig. S2 for sample profiles). A dose effect was observed in that the average percentage of IFN-γ positive CD4^+^ cells was 1.64%, 0.97%, and 0.51% in animals vaccinated with 100, 30, and 5 μg of −042, respectively, and 1.84%, 1.44%, and 0.44% in animals vaccinated with 100, 30, and 5 μg of −043 (Fig. 1e and f). There was a bias toward expression of Th1 cytokines IFN-γ and TNF-α, and responses were similar between the two vaccines. More than 5% of CD8^+^ T cells on average produced IFN-γ in response to restimulation with spike peptide pools for both vaccine constructs at all doses tested (Fig. 1f). These results showed induction of CD4^+^ and CD8^+^ T cell mediated immunity with a Th1-bias in mice vaccinated with GLB-COV2 mRNA vaccines.

### Immunogenicity in hamsters and protection from challenge with SARS-CoV-2 USA-WA1/2020. (i) SARS-CoV-2-specific antibodies.

**FIG 2:**
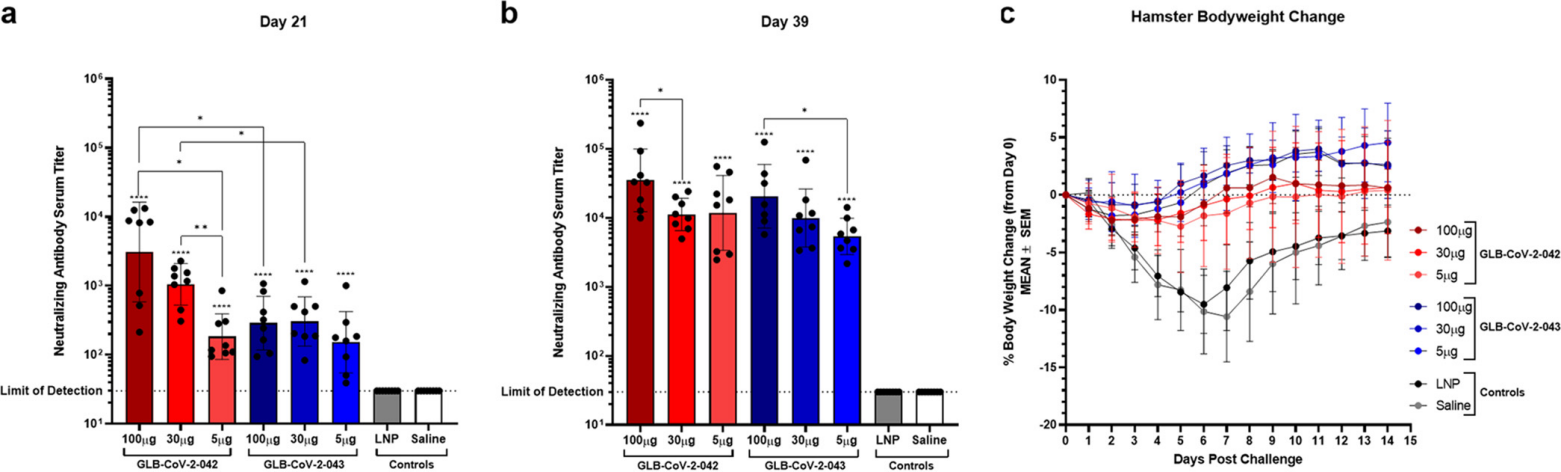
Immunogenicity in hamsters and protection from challenge with SARS-CoV-2 USA-WA1/2020. Golden Syrian Hamsters (*n* = 4 male and *n* = 4 female) were vaccinated with 100, 30, or 5 μg of GLB-COV2-042 or GLB-COV2-043 on days 0 and 21. (a and b) FRNT results on days 21 and 39. Means and standard deviations are shown. (c) Percent body weight change of each vaccination group over 14 days postinfection. Data from controls were combined for statistical analyses. Statistical analyses were performed using rank-based Mann-Whitney and Holm-Šidάk multiple-comparison tests. Statistical signifiers above bars represent significance between that group and controls. *, *P* < 0.05; ***, *P* < 0.005; ****, *P* < 0.001.

Immunogenicity and protection from SARS-CoV-2 challenge was next evaluated in the golden Syrian hamster model. Four male and four female hamsters per vaccine dose group were vaccinated with two doses of 100, 30, or 5 μg of −042 or −043 21 days apart. Serum Nab titers against the WA-1 strain of SARS-CoV-2 were quantified by FRNT (Fig. 2a and b). Immunization with one or two doses of 5, 30 or 100 μg of −042 or −043 significantly (*P* < 0.001) increased the serum neutralization GMT compared to mock-vaccinated or control animals that received LNP alone (Fig. 2a and b). At day 21, hamsters that received 100 or 30 μg of −042 had significantly higher (*P* < 0.05 and 0.005, respectively) Nab titers (GMTs of 3,110.16 and 1,050.54, respectively) than those that received 5 μg (GMT of 183.871) and these titers were also significantly higher than titers of the equivalent doses of −043 (GMTs of 288.581, 303.593, and 151.986 for 100 μg, 30 μg, and 5 μg, respectively) (Fig. 2a). 18 days after the second dose of −042, the Nab GMTs increased at all doses. The GMT after two doses of 100 μg −042 was significantly higher than the GMT of 30 μg (*P* < 0.05), but not 5 μg (*P* = 0.3518). Similarly, immunization with a second dose of 100, 30, and 5 μg of −043 increased the serum neutralizing GMTs to 20,576.22, 9,933.23, and 4,936.41, respectively. The GMT after two doses of 100 μg −043 was significantly higher than the GMT of 5 μg (*P* < 0.05), but not 30 μg (*P* = 0.3669). No significant differences in GMT between −042 and −043 were observed after two doses of the vaccine (Fig. 2b). These data demonstrated that both GLB-COV2 mRNA vaccines induced high titers of neutralizing anti-SARS-CoV-2 antibodies.

**(ii) Protection against homologous SARS-CoV-2 challenge.** Hamsters immunized twice with GLB-COV2-042 and −043 mRNA vaccines were challenged intranasally with 5.6 × 10^3^ PFU of WA-1 in 100 μL 21 days after the second immunization to evaluate the ability of GLB-COV2 mRNA vaccines to protect from homologous viral challenge. Mock and LNP immunized animals lost on average up to 10% of initial bodyweight by day 7 postchallenge before regaining weight. Two doses of −042 and −043 protected the hamsters from severe weight loss. Interestingly, no differences in weight loss were observed between hamsters that received two doses of 5, 30 or 100 μg mRNA vaccine. (Fig. 2c). −043 immunized animals gained weight more rapidly than −042 immunized animals, but all animals recovered their baseline bodyweight by day 9 postchallenge.

**FIG 3:**
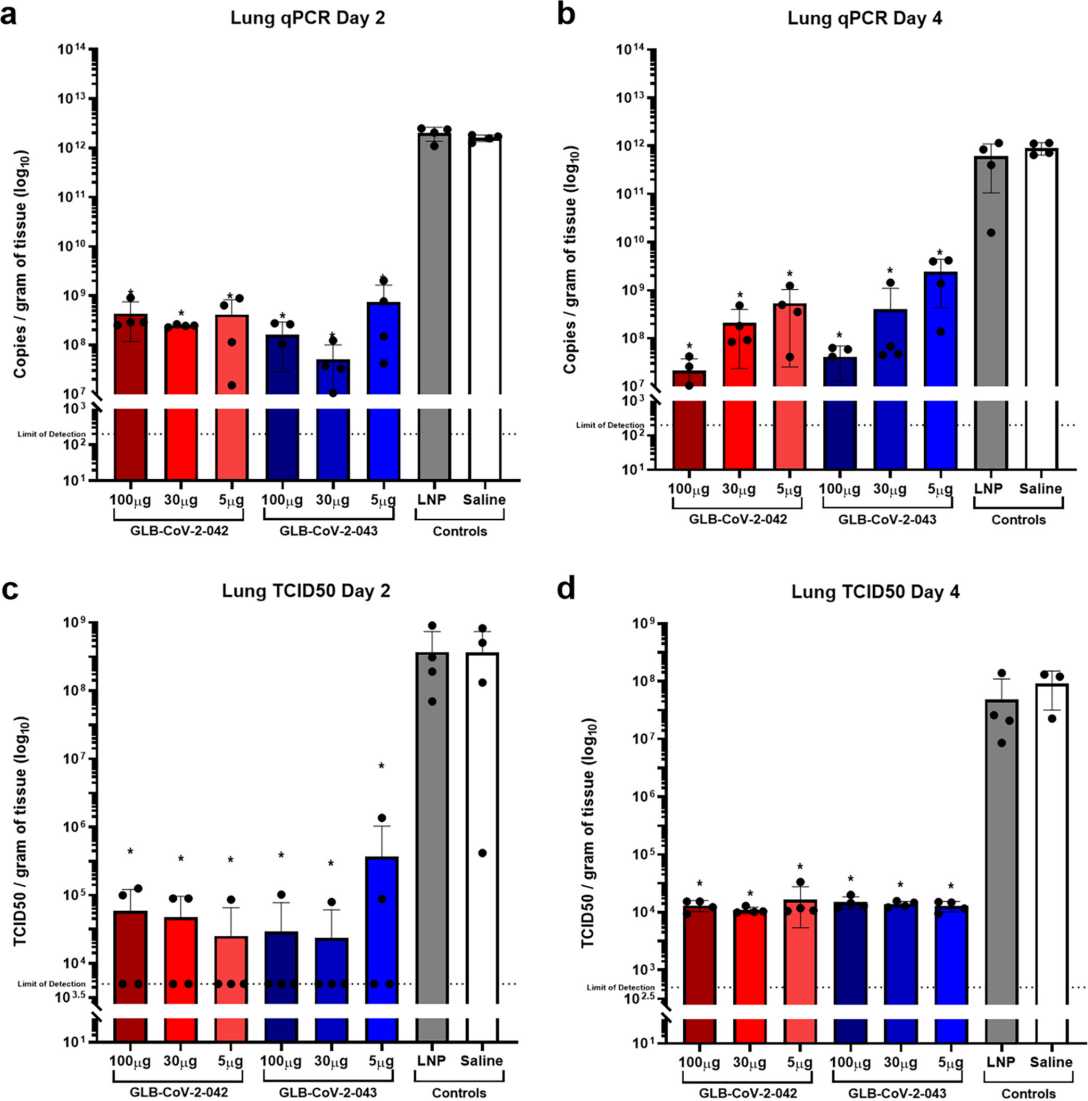
Quantification of SARS-CoV-2 in lungs of GLB-COV2 mRNA-vaccinated hamsters following SARS-CoV-2 challenge. Golden Syrian Hamsters (*n* = 4 male and *n* = 4 female) were vaccinated with 100, 30, or 5 μg of GLB-COV2-042 or GLB-COV2-043 on days 0 and 21 and challenges on day 42. (a and b) Detection of SARS-CoV-2 nucleocapsid via RT-qPCR at days 2 and 4 postchallenge. (c and d) Detection of active viral particles via TCID_50_ at days 2 and 4 postchallenge. Means and standard deviations are shown. Data from controls were combined for statistical analysis. Statistical analyses were performed using rank-based Mann-Whitney and Holm-Šidάk multiple-comparison tests. Statistical signifiers above bars represent significance between that group and controls. *, *P* < 0.05; ***, *P* < 0.005; ****, *P* < 0.001.

Next, we evaluated the effects of mRNA vaccination on infectious virus titers and viral RNA levels in the lungs and nasopharynx 2- and 4-days postchallenge. Viral RNA levels were quantified by RT-qPCR detecting the number of nucleocapsid (*N*) gene copies per gram of tissue. Infectious virus titers were determined by median tissue culture infectious dose (TCID_50_) assay and reported as TCID_50_/gram of tissue. The amount of viral RNA in the lungs of mock-vaccinated and LNP control vaccinated animals was 1× 10^12^ and 5× 10^11^ copies of *N* gene per gram of tissue 2 and 4 dpi (Fig. 3a to d). Immunization with two doses of 5, 30, and 100 μg of −042 reduced the number of *N* gene copies more than 1,000-fold at 2 dpi (Fig. 3a). No significant differences in the amount of viral RNA were observed between the three doses. Similar trends were seen in −043 immunized animals with reductions of over 1,000-fold at 2 dpi in all groups (Fig. 3a). No significant differences in *N* gene copies were found between equivalent doses of −042 and −043. These trends persisted at 4 dpi wherein *N* gene copy number remained 1,000-fold lower in all vaccinated animals than mock and LNP vaccinated animals with no significant differences between the three doses or −042 and −043 (Fig. 3b). Similarly, when viral replication was assessed at 2 and 4 dpi, TCID_50_ values of −042 and −043 vaccinated animals were over 1,000-fold lower than mock and LNP vaccinated animals (Fig. 3c). No differences between the three doses of each vaccine, or between equivalent doses of −042 and −043 were observed. By 4 dpi, viral titers in all −042 and −043 vaccinated animals had declined to at or near the limit of detection, while lung viral titers of mock and LNP controls remained at 1× 10^8^ (Fig. 3d). Together, these results demonstrated that GLB-COV2 vaccines effectively reduced viral replication in the lung of SARS-CoV-2 infected animals.

**FIG 4:**
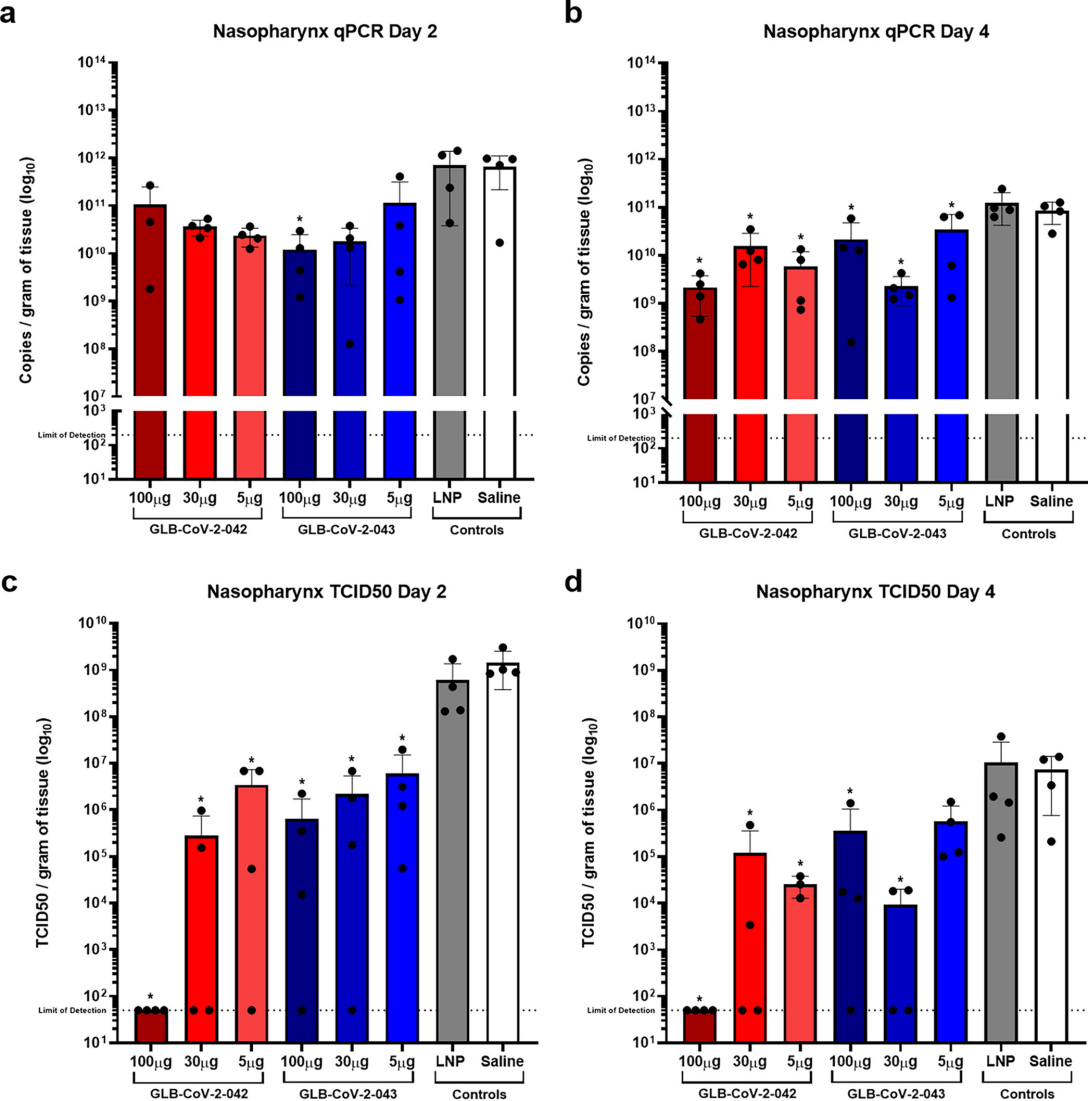
Quantification of SARS-CoV-2 in the nasopharynx of GLB-COV2 mRNA-vaccinated hamsters following viral challenge. Golden Syrian Hamsters (*n* = 4 male and *n* = 4 female) were vaccinated with 100, 30, or 5 μg of GLB-COV2-042 or GLB-COV2-043 on days 0 and 21 and challenged on day 42. (a and b) Detection of SARS-CoV-2 nucleocapsid via qPCR at days 2 and 4 postchallenge. (c and d) Detection of active viral particles via TCID_50_ at days 2 and 4 postchallenge. Means and standard deviations are shown. Data from controls were combined for statistical analysis. Statistical analyses were performed using rank-based Mann-Whitney and Holm-Šidάk multiple-comparison tests. Statistical signifiers above bars represent significance between that group and controls. *, *P* < 0.05; ***, *P* < 0.005; ****, *P* < 0.001.

Results from the nasopharynx with the same two assays are shown in Fig. 4a to d. Unlike the trend seen in the lung, there was no significant reduction in viral RNA levels in nasal washes at day 2 postchallenge (Fig. 4a). By day 4, the differences between vaccine and control groups reached significance, but RNA levels remained high in all groups (Fig. 4b). In contrast, significant reductions in infectious titers in nasal washes were seen in immunized animals compared to control groups at both days 2 and 4 postchallenge (Fig. 4c and d). Differences between vaccines and vaccine doses were minimal although within group variation was high, especially at day 4 postchallenge. Therefore, not only do GLB-COV2 vaccines limit viral replication in the lungs, but they are also effective at reducing SARS-CoV-2 replication in the nasopharynx.

To further evaluate the usefulness of GLB-CoV-2 vaccines in preventing or minimizing tissue damage from SARS-CoV-2 infection, lungs were taken from hamsters at days 2 and 4 postchallenge for histopathological analyses, as shown by Fig. S3 and Tables S1 and S2. At day 2 postchallenge, both GLB-COV2 vaccines demonstrated a lower incidence and severity of SARS-CoV-2 related findings in the lung in all groups compared to the LNP and saline control groups and no apparent differences between vaccines or doses were observed (Fig. S3, Table S1). At day 4 postchallenge, both −043 and −042 demonstrated a lower incidence and severity of SARS-CoV-2 related findings in the lung in all groups compared to the Saline and LNP control groups (Fig. S3; Table S2). Although both vaccines (mRNA Vaccine 1 and 2) had a similar decrease in severity and incidence of findings, −042 had an overall lower incidence and severity of findings compared to −043 at the 5 μg dose (Fig. S3; Table S2). At both time points vaccinated animals exhibited no to mild histological findings in the lung while all controls animals were found to have mild to severe tissue damage (Tables S1 and S2). Therefore, GLB-COV2 vaccines were able to successfully limit injury to the lung following SARS-CoV-2 challenge.

### GLB-COV2-043 protection from challenge with SARS-CoV-2 variants in hamsters.

**FIG 5:**
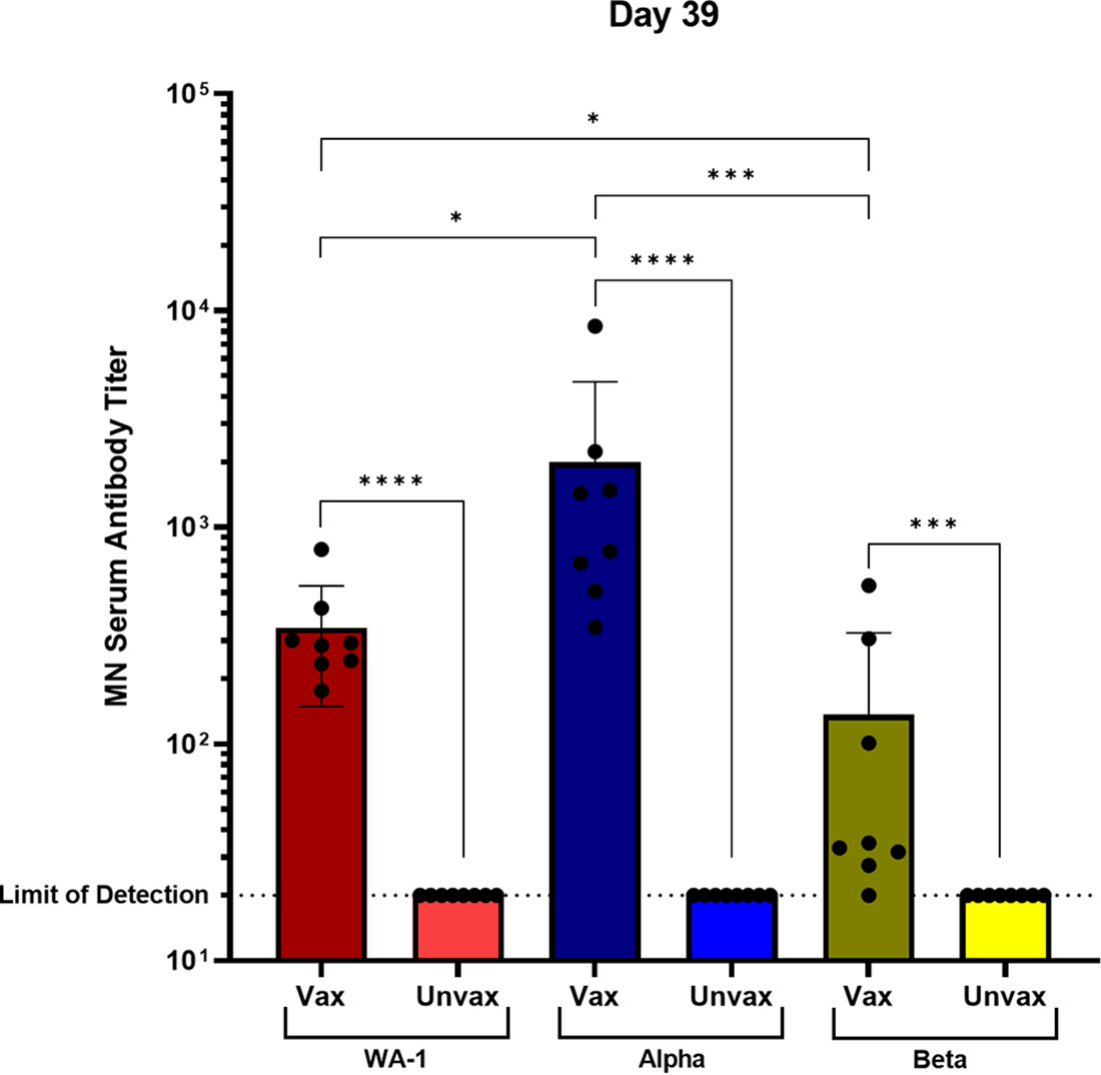
Neutralizing antibody response to WA-1 and variant SARS-CoV-2 viruses. Golden Syrian Hamsters (*n* = 8/group) were vaccinated with 30 μg of GLB-COV2-043 on days 0 and 21. Day 39 microneutralization titers in vaccinated and unvaccinated animals against each challenge virus are shown. Statistical analyses were performed using rank-based Mann-Whitney and Holm-Šidάk multiple-comparison tests. Statistical signifiers above bars represent significance between that group and controls. *, *P* < 0.05; ***, *P* < 0.005; ****, *P* < 0.001.

After demonstrating that the vaccines were protective against a homologous WA-1 challenge, we evaluated the ability of two doses of 30 μg of GLB-COV2-043 mRNA vaccine to protect against challenge with 10^6^ TCID_50_/mL of Alpha (B.1.1.7) and Beta (B.1.351) variants of SARS-CoV-2 in 100 μL (21). A homologous challenge with WA-1 was included as a control. Postboost antibody titers against each challenge virus were quantified using the microneutralization assay. When postboost titers against each virus were compared, vaccination with −043 induced Nab titers against Beta that were significantly lower than WA-1 and Alpha with GMTs of 65.4706, 309.299, and 1181.03 against Beta, WA-1, and Alpha, respectively. Interestingly, Nab titers against Alpha were significantly higher than those against homologous WA-1 (Fig. 5). Regardless, immunization with −043 resulted in robust titers against all challenge viruses, including Alpha and Beta variants, that were significantly higher than their respective controls when each virus was assayed (Fig. 5).

**FIG 6:**
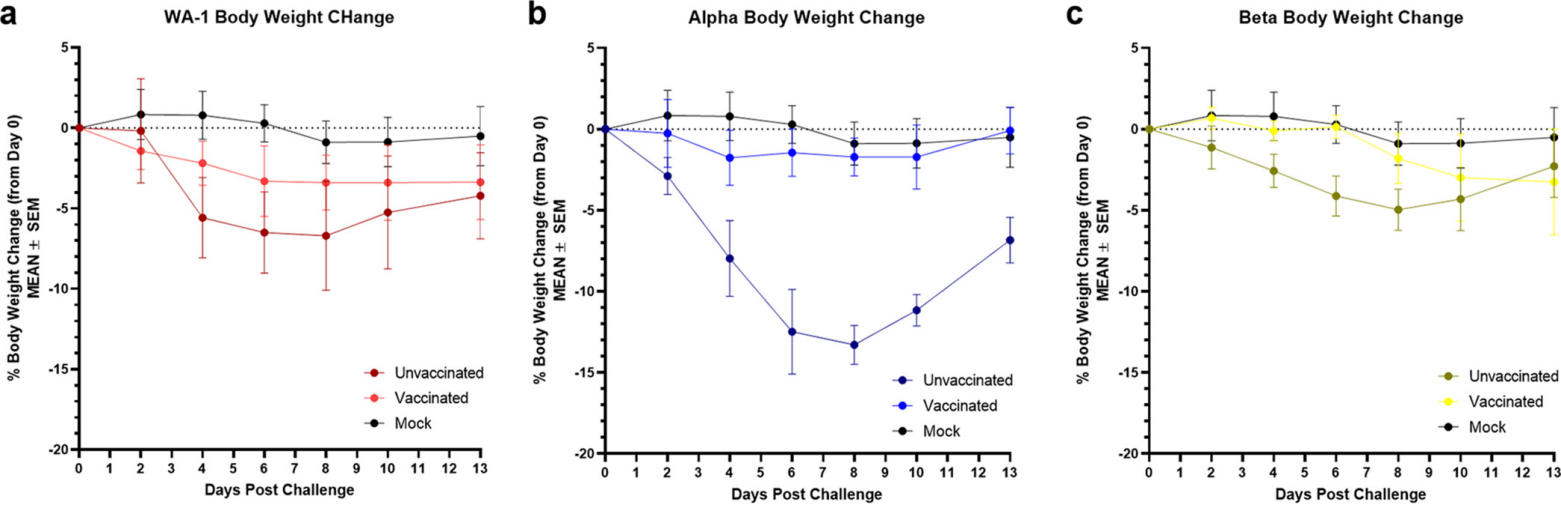
Morbidity in hamsters vaccinated with GLB-COV2 mRNA vaccines following SARS-CoV-2 variant challenge. Golden Syrian Hamsters (*n* = 8) were vaccinated with 30 μg of GLB-COV2-043 on days 0 and 21 and challenged on day 42. Percent body weight change of each vaccination group over 14 days postinfection.

When weight loss was assessed, unvaccinated age-matched control animals displayed variant-specific weight loss trends with Alpha animals losing the most weight (average 14% maximum weight loss) followed by WA-1 (average 7.2% maximum weight loss) then Beta (average 5.4% maximum weight loss) infected animals (Fig. 6a to c). Vaccinated animals lost less weight than their corresponding control groups with maximum average weight losses of 3.1%, 4.3%, and 3.4% in the Alpha, WA-1, and Beta challenge groups, respectively.

**FIG 7:**
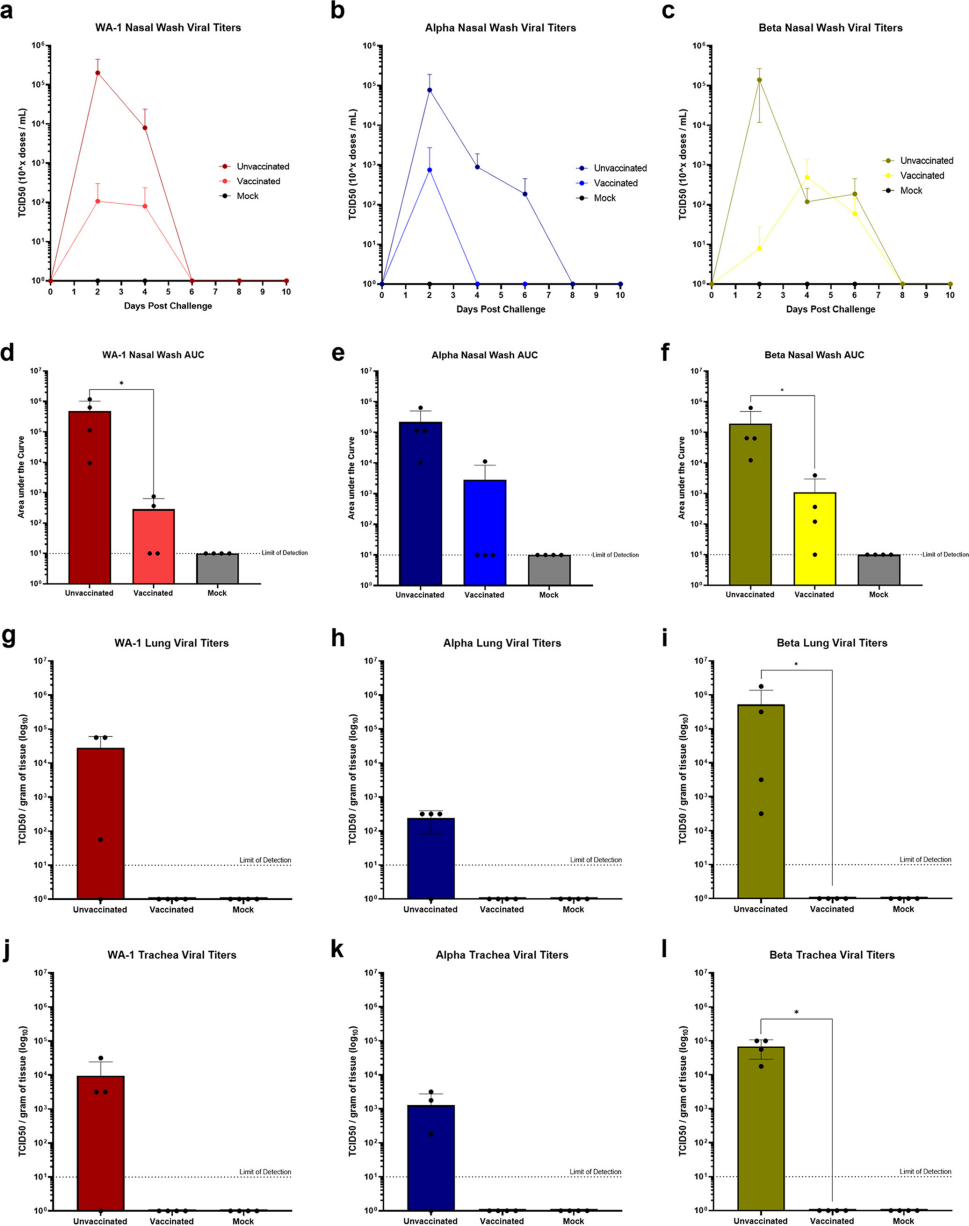
Viral titers in hamsters vaccinated with GLB-COV2 mRNA vaccines following SARS-CoV-2 variant challenge. Golden Syrian Hamsters (*n* = 8) were vaccinated with 30 μg of GLB-COV2-043 on days 0 and 21 and challenged on day 42. (a to c) Nasal wash TCID_50_ values over the course of infection. (d to f) Area under the curve analysis of nasal wash TCID_50_ values. (g-i) Lung TCID_50_ values 2 days postchallenge (*n* = 4). (j to l) Trachea TCID_50_ values 2 days postchallenge (*n* = 4). Means and standard deviations are shown. Data from controls were combined for statistical analysis. Statistical analyses were performed using rank-based Mann-Whitney and Holm-Šidάk multiple-comparison tests. Statistical signifiers above bars represent significance between that group and controls. *, *P* < 0.05; ***, *P* < 0.005; ****, *P* < 0.001.

We next evaluated the effect of −043 vaccination on viral replication in the upper and lower respiratory tracts of challenge animals. Nasal washes were taken on days 2, 4, 6, 8, and 10 postchallenge and lung and tracheal samples were harvested on day 2 postchallenge. TCID_50_ was performed to quantify infectious virus particles and results are shown in Fig. 7a to c. For all three viruses, viral loads in nasal washes and tissue samples were reduced postchallenge in vaccinated animals compared to unvaccinated. Following challenge with WA-1, unvaccinated and vaccinated animals had average maximum TCID_50_ values of 200,932.698 and 117.573 per mL of nasal wash, respectively (Fig. 7a). At 2 dpi, 8/8 unvaccinated animals were found to shed virus while only 4/8 vaccinated animals shed infectious virus particles. At 4 dpi infectious viral particles were detected by TCID_50_ in 2/4 unvaccinated and 1/4 remaining vaccinated animals. By 6 dpi no viral shedding was detected in any group. In the Alpha challenge group, average maximum TCID_50_ values of 77,316.02 and 750 per mL of nasal wash were found in the unvaccinated and vaccinated control groups, respectively (Fig. 7b). Notably, vaccinated animals shed no detectable virus by 4 dpi while unvaccinated animals continued to shed until 8 dpi. Differences in the number of infectious animals were also apparent at 2 dpi with 8/8 unvaccinated and 3/8 vaccinated animals positive for active viral replication by TCID_50_. Likewise, animals challenged with Beta had average maximum TCID_50_ values of 139,151.813 and 259 per mL of nasal wash in the unvaccinated and vaccinated groups, respectively (Fig. 7c). Animals infected with Beta shed virus until 8 dpi in both unvaccinated and vaccinated groups, though differences in the number of animals with infectious viral particles in the nasal wash were found at 2 (8/8 and 1/8 unvaccinated and vaccinated, respectively) and 4 (3/4 and 2/4 unvaccinated and vaccinated, respectively) dpi. Significant differences were observed in the nasal washes of vaccinated and unvaccinated animals challenged with WA-1 and Beta when under the curve analysis was performed (Fig. 7d and f). Strikingly, there were no infectious viral particles detected in the lungs or trachea of vaccinated animals in any of the virus challenge groups, and this reduction was statistically significant between vaccinated and unvaccinated animals challenged with Beta (Fig. 7g to l). These results demonstrated the protective effects of −043 in reducing viral burden caused by SARS-CoV-2 variants.

## DISCUSSION

This study is the first to evaluate GreenLight Biosciences’ mRNA vaccine for protection against SARS-CoV-2. GreenLight Biosciences previously developed a proprietary method for rapid and cost-effective RNA production. In this study, we have demonstrated that both GLB-COV2 mRNA vaccine constructs, −042 and −043, induce robust humoral and cellular responses against SARS-CoV-2 S1 antigens in the mouse model. Vaccination with −042 and −043 induced spike-specific neutralizing titers and elicited antigen-specific CD4^+^ and CD8^+^ T cell responses. Both candidates induced a clear Th1-skewed response, with preferential production and secretion of IFN-γ, TNF-α and IL-2 compared to the Th2-type cytokines, IL-4 and IL-5. Vaccine-associated enhanced respiratory disease has been described in some animal models following vaccination with whole-inactivated SARS-CoV (22), a response which was not observed in our animals. Following challenge with a virulent SARS-CoV-2 strain, hamsters vaccinated with −042 and −043 displayed significantly less weight loss than negative controls. Vaccination with −042 and −043 resulted in significantly reduced levels of viral RNA compared to controls at all stages of the disease model in both the nasopharynx and lung.

As mRNA vaccines enter the clinic, ensuring their safety and efficacy in humans is critical. Inclusion of modified nucleosides, namely, pseudouridine, in mRNA vaccines has been well-documented to increase translational efficiency of these constructs by reducing innate immune signaling and suppressing recognition of foreign dsRNA (23–28). Additionally, by dampening the innate immune response, the reactogenic profile of mRNA vaccines can be tempered; this strategy has been employed by both Moderna and Pfizer in the generation of their SARS-Cov-2 mRNA vaccines. Though both GLB-COV2 mRNA vaccines were comparable in immunogenicity and protection from viral challenge in both the mouse and hamster models, to ensure acceptable reactogenicity and efficacy, we chose to pursue candidate −043 in subsequent studies.

As SARS-CoV-2 continues to evolve with the emergence of new variants, vaccine breadth of protection must be investigated. Therefore, we assessed the utility of −043 in preventing or reducing disease caused by the Alpha and Beta variants. Studies evaluating animal and human convalescent and postimmunization sera support the notion that monoclonal and polyclonal antibodies to the original ancestral spike protein exert reduced neutralizing activity against these variants (29–31). Our findings show that −043 is effective in reducing morbidity caused by Alpha and Beta infection, as well as limiting viral replication in the respiratory tract as indicated by reduced viral titers in nasal wash and loss of detectable viral particles in the lung and trachea of vaccinated animals. However, this does not answer the question of effectiveness against variants that have emerged since the completion of these studies, such as Omicron. Antibodies isolated from convalescent and vaccinated individuals have demonstrated significantly reduced neutralizing activity against the Omicron variant, even those that were boosted with mRNA-based vaccines (32). Similarly, a study by Garcia-Beltran, et al. found sera from mRNA-vaccinated individuals exhibited low to undetectable levels of neutralization against Omicron. However, individuals that had been boosted did exhibit neutralizing activity against Omicron, albeit 4- to 6-fold lower than neutralization of wild-type SARS-CoV-2, suggesting additional mRNA vaccine doses enable cross-neutralization (33). More recently, evaluation of vaccine efficacy against the Omicron variant in England found two doses of COMIRNATY had an efficacy of 65.5% that drastically declined over time, reaching only 8.8% at 25 or more weeks. Administration of a booster dose of COMIRNATY increased efficacy to 67.2% before declining to 45.7% after 10 or more weeks (34). Further studies are needed to assess the protective efficacy of GLB-COV2-043 against these variants, as well as to determine their cross-neutralization capability and longevity, particularly after two versus three doses. Of note, all mRNA vaccines administered to date have encoded the original ancestral spike protein. It is unclear if the use of variant spike proteins for booster doses enhances antibody cross-neutralization.

### Conclusions.

GreenLight Biosciences’ vaccine candidates, GLB-COV2-042 and GLB-COV2-043, induced measurable neutralizing antibodies in two animal species. Antigen-specific cell-mediated immunity (CMI) with a mixed CD4^+^ and CD8^+^ response skewing toward a Th1 response were observed in mice. Lastly, hamsters developed measurable specific immune responses following vaccination, and showed protection from body-weight loss and PCR recovery of virus following intranasal challenge with a virulent SARS-CoV-2 clinical isolate as well as Alpha and Beta variants. The results demonstrate the effectiveness of GreenLight Biosciences’ SARS-CoV-2 mRNA vaccines in preclinical models and encourage rapid advancement of GLB-COV2-043 to clinical trials.

## MATERIALS AND METHODS

### GLB-COV2 mRNA transfection.

To prepare for Western blot, 293 T cells (ATCC) were seeded at 3 × 10^5^ cells/well in 24-well plates in Opti-MEM (Thermo Fisher Scientific 51985091), 10% fetal calf serum, 1% Antibiotic-Antimycotic containing penicillin (used at 100 units/mL), streptomycin (used at 100 μg/mL), Amphotericin B (used at 0.25 μg/mL) Gibco Cat. #15240-062). Cells were incubated overnight to ≈80% confluence. Cells were then transfected with two different mRNA quantities (500 ng or 1,500 ng) using Lipofectamine MessengerMax reagent (Thermo Fisher LMRNA001) following the manufacturer’s recommendations. At 48-h posttransfection, supernatants were removed and cell monolayers disrupted with 100 μL of 1× passive lysis buffer (Promega E194A). Insoluble debris was pelleted by centrifugation.

For ELISA, 293 T cells were seeded at 2 × 10^4^ cells/well in 96-well plates for overnight incubation, resulting in ~80% to 90% confluence. Transfection reagents and conditions were again as for the Lipofectamine MessengerMax reagent per manufacturer’s recommendations, but with 100 ng per well of RNA generated by GreenLight Biosciences or with commercially available *in vitro* transcription reactions. After overnight incubation, cell monolayers were used as targets in an ELISA.

### Western blot.

Western blot gels were run using the NuPAGE MES kit (Thermo Fisher Scientific NP0060). A total of 15 μL of insoluble debris posttransfection was resolved by SDS-PAGE under reducing conditions. Recombinant his-tagged SARS-CoV-2 spike S1 domain (cat. 40591-V08H, SinoBio) was loaded as a positive control. Proteins were blotted to nitrocellulose, blocked (Li-Cor 927–60001), and probed with a rabbit primary antibody that binds to SARS-CoV2 spike receptor binding domain (RBD) (1:2,000, SinoBio, cat. 40592-T62) followed by incubation with a secondary antibody (goat anti-rabbit horseradish peroxidase [HRP] 1:15,000, Jackson, Cat.# 111035003) before visualization.

### ELISA.

Following transfection, cell monolayers were used as targets in ELISA. Cells were prepared for ELISA by fixation with 100 μL acetone/PBS (80/20) for 1 min. Wells were air-dried, rehydrated with 200 μL PBS, and then blocked with 1% BSA in PBS for 30 min at 37°C. To perform ELISAs, monoclonal anti-spike antibody (SINO Biologicals, Cat. #40150-D001) was added for 30 min at 37°C, followed by washes and the addition of goat anti-human IgG H+L conjugated to horseradish peroxidase (Southern Biologicals, Cat. #2010-05) for 30 min, 37°C. Plates were developed with TMB substrate (KPL 5120-0047) and reactions were stopped with 1M phosphoric acid. Readings were at OD 450 nm. Three negative controls were included in the experiment: mock-transfected cells, a firefly luciferase mRNA, and an EGFP mRNA.

### SARS-CoV-2 virus propagation.

SARS-CoV-2 USA-WA1/2020 (GISAID: EPI_ISL_404895) challenge stock was prepared by propagating a seed stock in Vero E6 cells (ATCC CRL-1586). To prepare Alpha (GISAID: EPI_ISL_751801) and Beta (GISAID: EPI_ISL_678570) challenge stock, Vero E6 TMPRSS2 cells were seeded in a T75 tissue culture flask in growth media (DMEM Sigma D6429 4.5g/L glucose, l-glutamine, sodium bicarbonate, supplemented with 10% FCS and 1% Antibiotic-Antimycotic containing penicillin (used at 100 units/mL), streptomycin (used at 100 μg/mL), Amphotericin B (used at 0.25 μg/mL) Gibco Cat. #15240-062) to be ~90% confluent for infection. To infect, cells were washed 1× with PBS and inoculated with 100 μL of virus in 5 mL of infection media (DMEM Sigma D6429, supplemented with 2% FCS and 1% Antibiotic-Antimycotic Gibco 15240-062). The cells and virus were incubated for 30 min at 37°C and 5% CO_2_ after which an additional 15 mL of infection media was added to the cells. Cells were returned to the incubator until 90% cytopathic effect (CPE) was observed at which time the supernatant was collected. All viral stocks were tested for sterility using blood agar plates and aliquots were stored at –80°C. Viral titer was determined by TCID_50_. Viruses were sequence confirmed before use.

### Mouse models.

Eight-week-old female C57BL/6 mice were immunized with different doses of the GLB-COV2-042 and GLB-COV2-043 mRNA vaccines. Animals received a prime-boost series of two intramuscular injections, at days 0 and 21, containing 5, 30, or 100 μg in a volume of 50 μL per hind limb. Control animals received 50 μL of saline or LNP control in the hind limb at days 0 and 21. Serum samples were collected longitudinally to quantify spike protein-specific binding antibodies (by ELISA) and virus neutralizing antibody (NAb) titers (focus reduction neutralization test; FRNT). Approximately 3 months after the priming dose, animals were euthanized, spleens removed, and cellular immune responses characterized by restimulation of splenocyte cultures with SARS-CoV-2 spike peptides.

### Hamster models.

Six- to 8-week-old male and female hamsters were immunized with different doses of the GLB-COV2-042 and GLB-COV2-043 mRNA vaccines. Homologous challenge animals received a prime-boost series of two intramuscular injections, at days 0 and 21, containing 5, 30, or 100 μg in a volume of 50 μL per hind limb. Twenty-one days after receiving an initial vaccination with the 5 μg dose of −043, two cohoused female hamsters were found dead; however, necropsy of these animals found the deaths were unrelated to vaccination. This group subsequently had six animals. Control animals received 50 μL of saline or LNP control in the hind limb at days 0 and 21. Serum samples were collected longitudinally to quantify NAb titers. SARS-CoV-2 USA-WA1/2020 was administered intranasally to ketamine/xylazine-anesthetized animals (5.6 × 10^3^ PFU/100 μL). Dosing and blood sampling were performed under isoflurane anesthesia. Following challenge, hamsters were weighed daily. On days 44 and 46 postinitial immunization, animals were euthanized for lung and nasopharyngeal tissue collection and terminal bleed. For the variant challenges, vaccinated groups received 30 μg of GLB-COV2-043 on days 0 and 21. Unvaccinated animals served as the control group. 100 μL of virus at 10^6^ TCID_50_/mL was delivered intranasally, 50 μL per nostril, following isoflurane anesthesia on day 42 postimmunization. Mock challenged animals received 50 μL of sterile PBS per nostril. Hamsters were monitored for recovery. Nasal wash samples were collected on days 0, 2, 4, 6, 8, and 10 postinfection. Hamsters were anesthetized with 100 mg/kg ketamine hydrochloride delivered intraperitoneal (IP). 0.5 mL of sterile PBS was delivered intranasally using a 1 mL syringe fitted with an 18 gauge × 1.16 in. catheter, with a slow continuous drip into the left nostril and collected from the right nostril by gravity into a 4 oz specimen cup. The cups were centrifuged at 1,000 g for 2 min and the sample was transferred to a 1.8-mL Sarstedt tube and frozen at –80°C until titration. Day 2 postinfection, four animals from each group were necropsied for tissue sample. To collect lung and trachea, hamsters were anesthetized with continuous isoflurane through a nose cone during euthanasia. Each was euthanized with a barbiturate overdose using 500 μL Euthasol (100 mg/mL) administered intracardially. The following tissues were collected: ~1cm length of trachea, 0.5 cm^3^ from the lower left lung lobe, ~2cm length of small intestine, and 1 kidney, and placed into a 2.0 mL Safelock tube containing 500 μL of PBS and stored at –80°C until titration.

### Focus reduction neutralizing test (FRNT).

Approximately 24 h after plating Vero E6 cells, heat-inactivated sera (30 min at 56°C) was serially diluted at 1:5 for day 21 mouse and hamster sera, 1:3 for day 28 mouse sera, and 1:10 for day 39 hamster and day 42 mouse sera. One-hundred focus forming units (ffu) in 50 μL were added to each well and incubated at 37°C for 1 h. Tissue culture media were aspirated, 100 μL of “serum+virus” were added to each well, and plates incubated for 1 h at 37°C. After incubation, cells were washed with PBS, overlaid with 100 μL of 2% methylcellulose, and plates incubated for 24 h to 30 h at 37°C. After incubation, cells were fixed with 4% formalin for 20 to 30 min at room temperature, washed twice with PBS and then permeabilized (PBS with 0.5% Saponin and 2% FBS) for 15 to 30 min at room temperature. An anti-S monoclonal antibody (1C02, generously provided by Dr. Ali Ellebedy, Washington University) in permeabilization buffer was added (50 to 100 μL of 1:2,000 to 1:4,000 dilutions) to PBS washed cells and incubated for 1 h at room temperature with occasional rocking. The plates were washed 3 times with 0.05% Tween 20/PBS and a secondary detection antibody (anti-human IgG conjugated to HRP; 50 to 100 μL of 1:1,000 dilution) was added and incubated for 1 h at room temperature. Following three washes with 0.05% Tween 20/PBS, 3,3′,5,5′-tetramethylbenzidine (TMB) substrate (SK-4400 from Vector Laboratories) was added to develop spots, plates washed with water to stop the reaction, and foci counted.

### Microneutralization.

Five-fold serial dilutions were performed on heat-inactivated sera (1 h at 56°C) in infection media, starting at a 1:10 dilution. A standardized amount of infectious SARS-CoV-2 virus (250 TCID_50_), diluted in infection media, was added to the diluted serum at a 1:1 ratio and incubated for 1 h at 37°C. A total of 100 μL of the sera-virus mixture was added to VeroE6/TMPRSS2 cells seeded in 96-well plates the previous day and incubated for 1 h at 37°C and 5% CO2. Subsequently, an additional 100 μL of infection media was added and the cells incubated for a further 24 h to 48 h. Following incubation, cells were fixed with 4% formaldehyde (Polysciences Cat. #18814-20) for 30 min, washed with PBS three times, and then incubated with a block/permeabilization buffer (PBS supplemented with 3% Bovine Serum Albumin [BSA; Sigma-Aldrich Cat. #A8327-500 ml] and 0.2% Triton X-100 [Thermo Fisher Scientific Surfact-Amps-X-100, 10% Solution Cat. #28314]) for 30 min. Rabbit anti-SARS CoV-2 NP MAb (Sinobiologicals Cat. #40143-R040) at a 1:2,000 dilution was added for 1 h. Cells were washed three times with PBS + 0.5% Tween before incubation with a secondary goat anti-rabbit IgG–HRP conjugated antibody (Cell Signaling Cat. #7074S) at a 1:3,000 dilution for 1 h. After washing the cells 3 times with PBST, 100 μL of TMB (Thermo Fisher Scientific Cat. #N301) was added and color developed for 10 min before 1N sulfuric acid (Thermo Fisher Scientific Cat. #SA212-1) was added to stop the reaction. The optical density was measured at 450 nm on a Biotek Synergy plate microplate reader and the neutralization titers were calculated as the reciprocal serum dilution (IC_50_) causing 50% reduction of relative light units.

### T cell analyses.

Five mice from each vaccine dose group (100, 30, or 5 μg) including −042 and −043 vaccinated groups, three saline controls, and four LNP controls were sacrificed approximately 3 months postpriming vaccine. Spleens were removed and single cell suspensions were prepared. Red blood cells were lysed, and white blood cells were purified by centrifugation on ficoll gradients. Cells were counted, adjusted to 1 × 10^6^ cells/well and cultured in a 96-well plate in RPMI media supplemented with 5% fetal calf serum (FCS), glutamine, gentamicin (0.5 mg/mL) and 5 × 10^−5^ M 2-mercaptoethanol. Cells were stimulated with approximately 0.5 μg/mL of a pool of 253 unique, overlapping 15 mer peptides, representing the SARS-CoV-2 spike protein (peptides were from Mimotopes, Mulgrave, Victoria, Australia). Splenocytes were plated separately for (i) analyses of secreted cytokines, and (ii) intracellular cytokine (ICC) assays.

For tests of secreted cytokines, cells were plated in round-bottomed plates and supernatants were collected after overnight incubation. The supernatants were assayed using a Multiplex kit (Millipore MAP Kit) with a Luminex 200 Multiplexing Instrument and xPonent software. Results from wells with unstimulated cells were subtracted from test results.

For ICC assays, splenocytes were plated in flat bottomed plates, treated with Brefeldin A and incubated for 6 h. After incubation, cells were washed and blocked with anti-CD16/32 (FcBlock). Cells were then stained for surface markers CD3 (BV711-labeled antibodies), CD4 (APC-labeled antibodies), and CD8 (APC Cy7-labeled antibodies). Cells were next fixed and permeabilized, after which they were stained for the presence of intracellular cytokines with antibodies specific for IFN-γ (FITC-labeled antibodies), TNF-α (PE Dazzle 594-labeled antibodies), IL-2 (PE-labeled antibodies), IL-4 (BV605-labled antibodies), and IL-5 (BV421-labeled antibodies). Cells were washed and prepared for analysis on a Fortessa LSR flow cytometer (BD Biosciences). Data were analyzed using FCS Express software.

### Nucleocapsid qPCR.

Viral RNA copy numbers were determined using a RT-qPCR assay. Viral RNA was extracted from nasal wash using the Qiagen MinElute virus spin kit (cat. no. 57704). RNA from tissues were extracted with RNA-STAT 60 (Tel-test “B”)/chloroform, precipitated and resuspended in RNase-free water. Controls were generated by isolating RNA from the applicable SARS-CoV-2 stock using the same procedure. The amount of RNA was determined from an OD reading at 260 and the number of copies was then calculated and a final dilution of 10^8^ copies/3 μL was stored at –80°C until needed. The master mix was prepared with 2.5 mL of 2× buffer containing Taq-polymerase, 50 μL of the reverse transcriptase and 100 μL of RNase inhibitor (TaqMan RT-PCR kit; Bioline Cat.#BIO-78005), 1.5 mL of the 2 μM primer pair, 0.5 mL of water and 350 μL of the 2 μM probe. For the reactions, 45 μL of the master mix and 5 μL of the sample RNA was added to a 96-well plate and all samples run in triplicate. A standard curve was prepared using the control RNA to provide a range of 1 to 10^7^ RNA copies/reaction and run in duplicate. The plate was placed in an Applied Biosystems 7500 Sequence detector and amplified using the following program: 48°C for 30 min, 95°C for 10 min followed by 40 cycles of 95°C for 15 s, and 1 min at 55°C. The number of copies of RNA per mL was calculated by extrapolation from the standard curve and multiplying by the reciprocal of 0.2 mL extraction volume.

Primers/probe sequences:

2019-nCoV_N1-F: 5′-GAC CCC AAA ATC AGC GAA AT-3′

2019-nCoV_N1-R: 5′-TCT GGT TAC TGC CAG TTG AAT CTG-3′

2019-nCoV_N1-P: 5′-FAM-ACC CCG CAT TAC GTT TGG TGG ACC-BHQ1-3′

### TCID_50_ of SARS-CoV-2.

To assay lung and nasopharynx samples following homologous SARS-CoV-2 challenge, Vero E6 cells (ATCC Cat. #CRL-1586) were plated in 96-well flat-bottom plates in DMEM (Thermo Fisher Scientific, Cat. #10566-016) with 10% FBS+gentamicin (gentamicin concentration of 1 mg/mL; Quality Biological Cat. #120-099-661) and incubated overnight at 37°C at 5% CO_2_ until 80% to 100% confluent. Media was aspirated and replaced with 180 μL of DMEM + 2% FBS+gentamicin (gentamicin concentration of 0.2 mg/mL). A total of 20 μL of each test sample was added to the first row of wells in quadruplicate to create 1:10 dilutions and log_10_ dilutions were performed across each plate. After 4 days, the cells were visually inspected for CPE and TCID_50_ were calculated using the Reed-Muench formula (35). To assay nasal wash, lung, and trachea following variant challenge, Vero E6 TMPRSS2 cells were plated in 96-well flatbottom tissue culture plates, to be near 80% confluence the following day. Samples were titrated eight logs starting at a 1:10 dilution in infection media. Cells were washed with infection media and sample dilutions were added in triplicate and plates incubated at 37°C, 5% CO_2_. After 72 h, the supernatant was removed, and cells were fixed and stained with 0.1% crystal violet in 10% formaldehyde for 20 min. Plates were scored for infectivity and the TCID_50_ was calculated by the method described in Reed and Muench (35).

### Histopathology.

Animals from each vaccine and control group that were selected for necropsy were submitted on days 2 and 4 postchallenge. Lungs were collected and transferred directly to formalin and sent to Charles River Laboratories Inc., Frederick, MD where they were trimmed, processed routinely, embedded in paraffin, and stained with H&E. Light microscopic evaluation was conducted by the Study Pathologist, a board-certified veterinary pathologist on lungs.

### Statistical tests.

Statistical tests were performed with GraphPad Prism Software. OD_450_ values were plotted in using nonlinear regression to determine binding antibody endpoint; nonlinear regression was also used to calculate IC_50_ values of neutralizing antibodies. Rank-based Mann-Whitney was performed with Holm-Šidάk correction to account for multiple comparisons.

## References

[1] Johns Hopkins University. 2021. COVID-19 dashboard, on Johns Hopkins University of Medicine. https://coronavirus.jhu.edu/map.html.

[2] Dong E, Du H, Gardner L. 2020. An interactive web-based dashboard to track COVID-19 in real time. Lancet Infect Dis 20:533–534. doi:10.1016/S1473-3099(20)30120-1. 32087114 PMC7159018

[3] Anonymous. 2021. Comirnaty and Pfizer-BioNTech COVID-19 vaccine, on U. S. Food and Drug Administration. https://www.fda.gov/emergency-preparedness-and-response/coronavirus-disease-2019-covid-19/comirnaty-and-pfizer-biontech-covid-19-vaccine.

[4] Anonymous. 2021. Moderna COVID-19 vaccine, on U. S. Food and Drug Administration. https://www.fda.gov/emergency-preparedness-and-response/coronavirus-disease-2019-covid-19/moderna-covid-19-vaccine.

[5] Anonymous. 2021. Janssen COVID-19 vaccine, on U. S. Food and Drug Administration. https://www.fda.gov/emergency-preparedness-and-response/coronavirus-disease-2019-covid-19/janssen-covid-19-vaccine.

[6] Polack FP, Thomas SJ, Kitchin N, Absalon J, Gurtman A, Lockhart S, Perez JL, Pérez Marc G, Moreira ED, Zerbini C, Bailey R, Swanson KA, Roychoudhury S, Koury K, Li P, Kalina WV, Cooper D, Frenck RW, Jr, Hammitt LL, Türeci Ö, Nell H, Schaefer A, Ünal S, Tresnan DB, Mather S, Dormitzer PR, Şahin U, Jansen KU, Gruber WC, C4591001 Clinical Trial Group. 2020. Safety and efficacy of the BNT162b2 mRNA COVID-19 vaccine. N Engl J Med 383:2603–2615. doi:10.1056/NEJMoa2034577. 33301246 PMC7745181

[7] Baden LR, El Sahly HM, Essink B, Kotloff K, Frey S, Novak R, Diemert D, Spector SA, Rouphael N, Creech CB, McGettigan J, Khetan S, Segall N, Solis J, Brosz A, Fierro C, Schwartz H, Neuzil K, Corey L, Gilbert P, Janes H, Follmann D, Marovich M, Mascola J, Polakowski L, Ledgerwood J, Graham BS, Bennett H, Pajon R, Knightly C, Leav B, Deng W, Zhou H, Han S, Ivarsson M, Miller J, Zaks T, COVE Study Group. 2021. Efficacy and safety of the mRNA-1273 SARS-CoV-2 vaccine. N Engl J Med 384:403–416. doi:10.1056/NEJMoa2035389. 33378609 PMC7787219

[8] Bollyky TJ. 2021. U.S. COVID-19 vaccination challenges go beyond supply. Ann Intern Med 174:558–559. doi:10.7326/M20-8280. 33395338 PMC7808440

[9] Abu-Raddad LJ, Chemaitelly H, Butt AA, National Study Group for COVID-19 Vaccination. 2021. Effectiveness of the BNT162b2 COVID-19 vaccine against the B.1.1.7 and B.1.351 variants. N Engl J Med 385:187–189. doi:10.1056/NEJMc2104974. 33951357 PMC8117967

[10] Bruxvoort KJ, Sy LS, Qian L, Ackerson BK, Luo Y, Lee GS, Tian Y, Florea A, Aragones M, Tubert JE, Takhar HS, Ku JH, Paila YD, Talarico CA, Tseng HF. 2021. Effectiveness of mRNA-1273 against delta, mu, and other emerging variants of SARS-CoV-2: test negative case-control study. BMJ 375:e068848. doi:10.1136/bmj-2021-068848. 34911691 PMC8671836

[11] Tauzin A, Nayrac M, Benlarbi M, Gong SY, Gasser R, Beaudoin-Bussières G, Brassard N, Laumaea A, Vézina D, Prévost J, Anand SP, Bourassa C, Gendron-Lepage G, Medjahed H, Goyette G, Niessl J, Tastet O, Gokool L, Morrisseau C, Arlotto P, Stamatatos L, McGuire AT, Larochelle C, Uchil P, Lu M, Mothes W, Serres GD, Moreira S, Roger M, Richard J, Martel-Laferrière V, Duerr R, Tremblay C, Kaufmann DE, Finzi A. 2021. A single BNT162b2 mRNA dose elicits antibodies with Fc-mediated effector functions and boost pre-existing humoral and T cell responses. bioRxiv. doi:10.1101/2021.03.18.435972. PMC8175625 34133950

[12] Pardi N, Hogan MJ, Porter FW, Weissman D. 2018. mRNA vaccines - a new era in vaccinology. Nat Rev Drug Discov 17:261–279. doi:10.1038/nrd.2017.243. 29326426 PMC5906799

[13] Petsch B, Schnee M, Vogel AB, Lange E, Hoffmann B, Voss D, Schlake T, Thess A, Kallen KJ, Stitz L, Kramps T. 2012. Protective efficacy of in vitro synthesized, specific mRNA vaccines against influenza A virus infection. Nat Biotechnol 30:1210–1216. doi:10.1038/nbt.2436. 23159882

[14] Zhuang X, Qi Y, Wang M, Yu N, Nan F, Zhang H, Tian M, Li C, Lu H, Jin N. 2020. mRNA vaccines encoding the HA protein of influenza A H1N1 virus delivered by cationic lipid nanoparticles induce protective immune responses in mice. Vaccines (Basel) 8. doi:10.3390/vaccines8010123. PMC7157730 32164372

[15] Richner JM, Himansu S, Dowd KA, Butler SL, Salazar V, Fox JM, Julander JG, Tang WW, Shresta S, Pierson TC, Ciaramella G, Diamond MS. 2017. Modified mRNA vaccines protect against Zika virus infection. Cell 168:1114–1125.e10. doi:10.1016/j.cell.2017.02.017. 28222903 PMC5388441

[16] Richner JM, Jagger BW, Shan C, Fontes CR, Dowd KA, Cao B, Himansu S, Caine EA, Nunes BTD, Medeiros DBA, Muruato AE, Foreman BM, Luo H, Wang T, Barrett AD, Weaver SC, Vasconcelos PFC, Rossi SL, Ciaramella G, Mysorekar IU, Pierson TC, Shi PY, Diamond MS. 2017. Vaccine mediated protection against Zika virus-induced congenital disease. Cell 170:273–283.e12. doi:10.1016/j.cell.2017.06.040. 28708997 PMC5546158

[17] Schnee M, Vogel AB, Voss D, Petsch B, Baumhof P, Kramps T, Stitz L. 2016. An mRNA vaccine encoding rabies virus glycoprotein induces protection against lethal infection in mice and correlates of protection in adult and newborn pigs. PLoS Negl Trop Dis 10:e0004746. doi:10.1371/journal.pntd.0004746. 27336830 PMC4918980

[18] Stitz L, Vogel A, Schnee M, Voss D, Rauch S, Mutzke T, Ketterer T, Kramps T, Petsch B. 2017. A thermostable messenger RNA based vaccine against rabies. PLoS Negl Trop Dis 11:e0006108. doi:10.1371/journal.pntd.0006108. 29216187 PMC5737050

[19] Pardi N, LaBranche CC, Ferrari G, Cain DW, Tombácz I, Parks RJ, Muramatsu H, Mui BL, Tam YK, Karikó K, Polacino P, Barbosa CJ, Madden TD, Hope MJ, Haynes BF, Montefiori DC, Hu SL, Weissman D. 2019. Characterization of HIV-1 nucleoside-modified mRNA vaccines in rabbits and Rhesus Macaques. Mol Ther Nucleic Acids 15:36–47. doi:10.1016/j.omtn.2019.03.003. 30974332 PMC6454128

[20] Saravia J, Chapman NM, Chi H. 2019. Helper T cell differentiation. Cell Mol Immunol 16:634–643. doi:10.1038/s41423-019-0220-6. 30867582 PMC6804569

[21] Anonymous. 2021. Science brief: emerging SARS-CoV-2 variants, on Centers for Disease Control and Prevention. https://www.cdc.gov/coronavirus/2019-ncov/science/science-briefs/scientific-brief-emerging-variants.html. 34009774

[22] Bolles M, Deming D, Long K, Agnihothram S, Whitmore A, Ferris M, Funkhouser W, Gralinski L, Totura A, Heise M, Baric RS. 2011. A double-inactivated severe acute respiratory syndrome coronavirus vaccine provides incomplete protection in mice and induces increased eosinophilic proinflammatory pulmonary response upon challenge. J Virol 85:12201–12215. doi:10.1128/JVI.06048-11. 21937658 PMC3209347

[23] Karikó K, Muramatsu H, Welsh FA, Ludwig J, Kato H, Akira S, Weissman D. 2008. Incorporation of pseudouridine into mRNA yields superior nonimmunogenic vector with increased translational capacity and biological stability. Mol Ther 16:1833–1840. doi:10.1038/mt.2008.200. 18797453 PMC2775451

[24] Andries O, Mc Cafferty S, De Smedt SC, Weiss R, Sanders NN, Kitada T. 2015. N(1)-methylpseudouridine-incorporated mRNA outperforms pseudouridine-incorporated mRNA by providing enhanced protein expression and reduced immunogenicity in mammalian cell lines and mice. J Control Release 217:337–344. doi:10.1016/j.jconrel.2015.08.051. 26342664

[25] Anderson BR, Muramatsu H, Jha BK, Silverman RH, Weissman D, Karikó K. 2011. Nucleoside modifications in RNA limit activation of 2'-5'-oligoadenylate synthetase and increase resistance to cleavage by RNase L. Nucleic Acids Res 39:9329–9338. doi:10.1093/nar/gkr586. 21813458 PMC3241635

[26] Anderson BR, Muramatsu H, Nallagatla SR, Bevilacqua PC, Sansing LH, Weissman D, Karikó K. 2010. Incorporation of pseudouridine into mRNA enhances translation by diminishing PKR activation. Nucleic Acids Res 38:5884–5892. doi:10.1093/nar/gkq347. 20457754 PMC2943593

[27] Karikó K, Buckstein M, Ni H, Weissman D. 2005. Suppression of RNA recognition by Toll-like receptors: the impact of nucleoside modification and the evolutionary origin of RNA. Immunity 23:165–175. doi:10.1016/j.immuni.2005.06.008. 16111635

[28] Nelson J, Sorensen EW, Mintri S, Rabideau AE, Zheng W, Besin G, Khatwani N, Su SV, Miracco EJ, Issa WJ, Hoge S, Stanton MG, Joyal JL. 2020. Impact of mRNA chemistry and manufacturing process on innate immune activation. Sci Adv 6:eaaz6893. doi:10.1126/sciadv.aaz6893. 32637598 PMC7314518

[29] Chen RE, Zhang X, Case JB, Winkler ES, Liu Y, VanBlargan LA, Liu J, Errico JM, Xie X, Suryadevara N, Gilchuk P, Zost SJ, Tahan S, Droit L, Turner JS, Kim W, Schmitz AJ, Thapa M, Wang D, Boon ACM, Presti RM, O'Halloran JA, Kim AHJ, Deepak P, Pinto D, Fremont DH, Crowe JE, Jr, Corti D, Virgin HW, Ellebedy AH, Shi PY, Diamond MS. 2021. Resistance of SARS-CoV-2 variants to neutralization by monoclonal and serum-derived polyclonal antibodies. Nat Med 27:717–726. doi:10.1038/s41591-021-01294-w. 33664494 PMC8058618

[30] Zhou D, Dejnirattisai W, Supasa P, Liu C, Mentzer AJ, Ginn HM, Zhao Y, Duyvesteyn HME, Tuekprakhon A, Nutalai R, Wang B, Paesen GC, Lopez-Camacho C, Slon-Campos J, Hallis B, Coombes N, Bewley K, Charlton S, Walter TS, Skelly D, Lumley SF, Dold C, Levin R, Dong T, Pollard AJ, Knight JC, Crook D, Lambe T, Clutterbuck E, Bibi S, Flaxman A, Bittaye M, Belij-Rammerstorfer S, Gilbert S, James W, Carroll MW, Klenerman P, Barnes E, Dunachie SJ, Fry EE, Mongkolsapaya J, Ren J, Stuart DI, Screaton GR. 2021. Evidence of escape of SARS-CoV-2 variant B.1.351 from natural and vaccine-induced sera. Cell 184:2348–2361.e6. doi:10.1016/j.cell.2021.02.037. 33730597 PMC7901269

[31] Wang P, Nair MS, Liu L, Iketani S, Luo Y, Guo Y, Wang M, Yu J, Zhang B, Kwong PD, Graham BS, Mascola JR, Chang JY, Yin MT, Sobieszczyk M, Kyratsous CA, Shapiro L, Sheng Z, Huang Y, Ho DD. 2021. Antibody resistance of SARS-CoV-2 variants B.1.351 and B.1.1.7. Nature 593:130–135. doi:10.1038/s41586-021-03398-2. 33684923

[32] Liu L, Iketani S, Guo Y, Chan JF, Wang M, Liu L, Luo Y, Chu H, Huang Y, Nair MS, Yu J, Chik KK, Yuen TT, Yoon C, To KK, Chen H, Yin MT, Sobieszczyk ME, Huang Y, Wang HH, Sheng Z, Yuen KY, Ho DD. 2021. Striking antibody evasion manifested by the omicron variant of SARS-CoV-2. Nature 602:676–681. doi:10.1038/s41586-021-04388-0. 35016198

[33] Garcia-Beltran WF, St. Denis KJ, Hoelzemer A, Lam EC, Nitido AD, Sheehan ML, Berrios C, Ofoman O, Chang CC, Hauser BM, Feldman J, Roederer AL, Gregory DJ, Poznansky MC, Schmidt AG, Iafrate AJ, Naranbhai V, Balazs AB. 2022. mRNA-based COVID-19 vaccine boosters induce neutralizing immunity against SARS-CoV-2 Omicron variant. Cell 185:457–466. doi:10.1016/j.cell.2021.12.033. 34995482 PMC8733787

[34] Andrews N, Stowe J, Kirsebom F, Toffa S, Rickeard T, Gallagher E, Gower C, Kall M, Groves N, O'Connell AM, Simons D, Blomquist PB, Zaidi A, Nash S, Iwani Binti Abdul Aziz N, Thelwall S, Dabrera G, Myers R, Amirthalingam G, Gharbia S, Barrett JC, Elson R, Ladhani SN, Ferguson N, Zambon M, Campbell CNJ, Brown K, Hopkins S, Chand M, Ramsay M, Lopez Bernal J. 2022. Covid-19 vaccine effectiveness against the omicron (B.1.1.529) variant. N Engl J Med 386:1532–1546. doi:10.1056/NEJMoa2119451. 35249272 PMC8908811

[35] Reed LJ, Muench H. 1938. A simple method of estimating fifty per cent endpoints. American J Epidemiology 27:5.

