# Supplementary material for: mRNA Vaccine Mitigates SARS-CoV-2 Infections and COVID-19
Source: Microbiol Spectr. 2023 Jan 25;11(1):e04240-22. doi: 10.1128/spectrum.04240-22 (PMC9927305; doi:10.1128/spectrum.04240-22)
Supplement: Supplemental file 1 — Supplemental material. Download spectrum.04240-22-s0001.pdf, PDF file, 0.6 MB [file spectrum.04240-22-s0001.pdf]

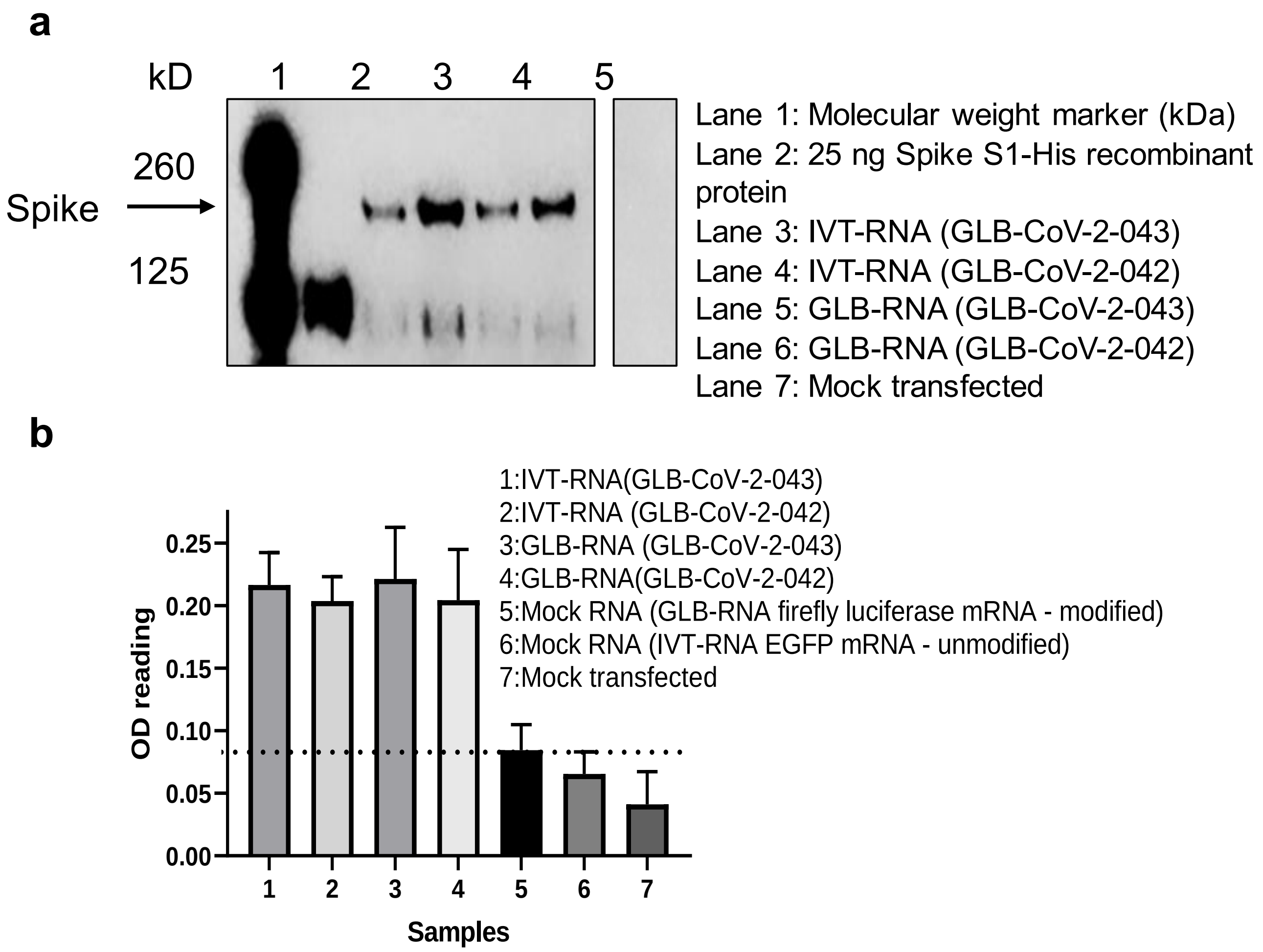

Supplementary Figure 1: In vitro Expression of GLB-COV2 mRNA 2

a) Western blot of 48-hour post transfection 239T cell extracts transfected with 500 ng of the listed mRNAs. Recombinant S1 was used as a control (Lane 2). b) ELISA using 24-hour post-transfection 293T cell extracts 4 transfected with the listed mRNAs.

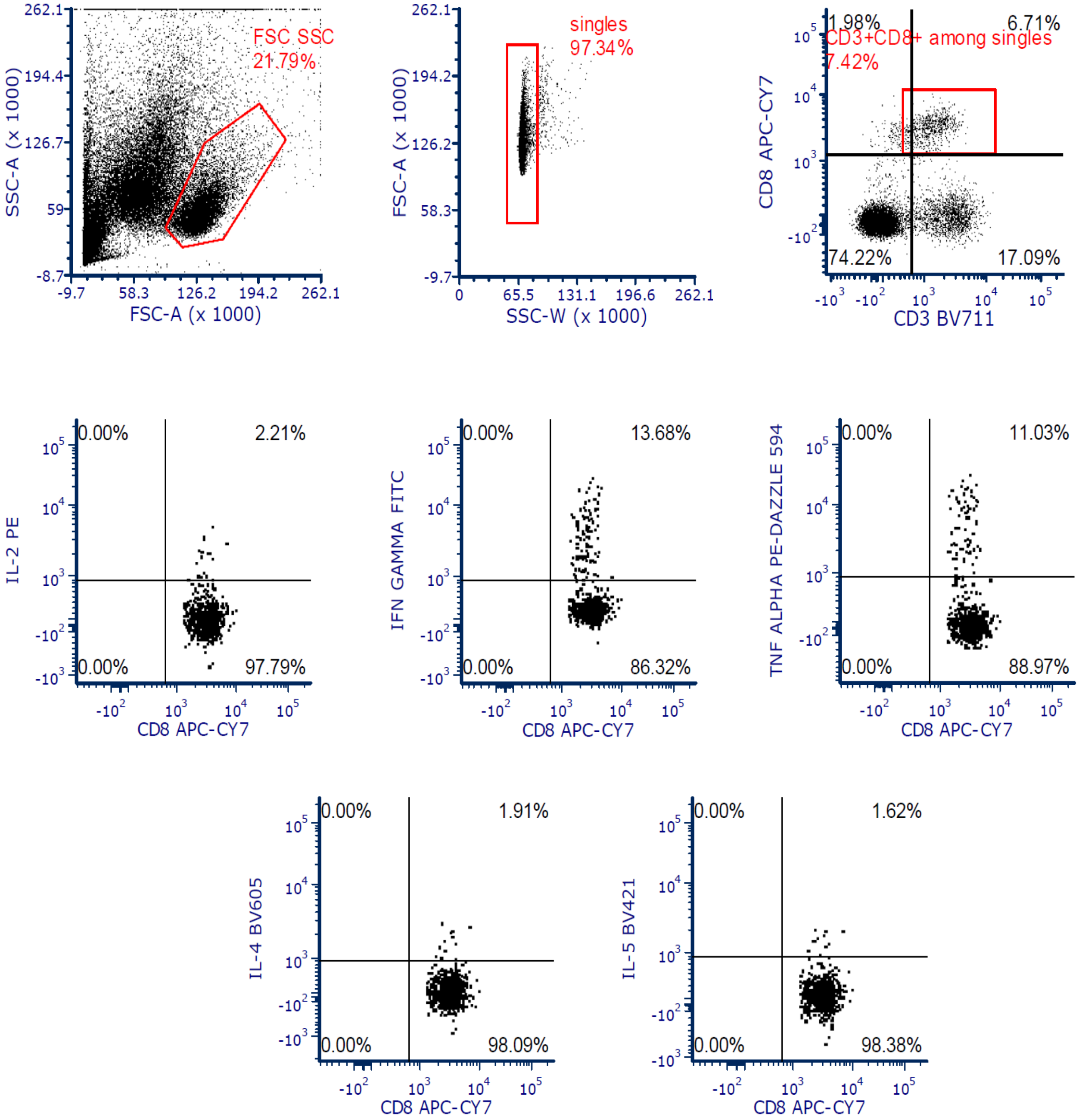

Supplementary Figure 2: Flow Cytometry Gating Strategy for Intracellular Cytokine Analysis

a) Sampling of flow cytometry profiles is shown. Lymphoid cells were first gated based on scatter profiles, after which CD3+CD8+ and CD3+CD4+populations were selected (see top row for CD3+CD8+ gating). Among CD3+CD8+ or CD3+CD4+populations, the

percentages of cells expressing different cytokines were determined. Profiles demonstrate gating strategies and marker positions for the various stains.

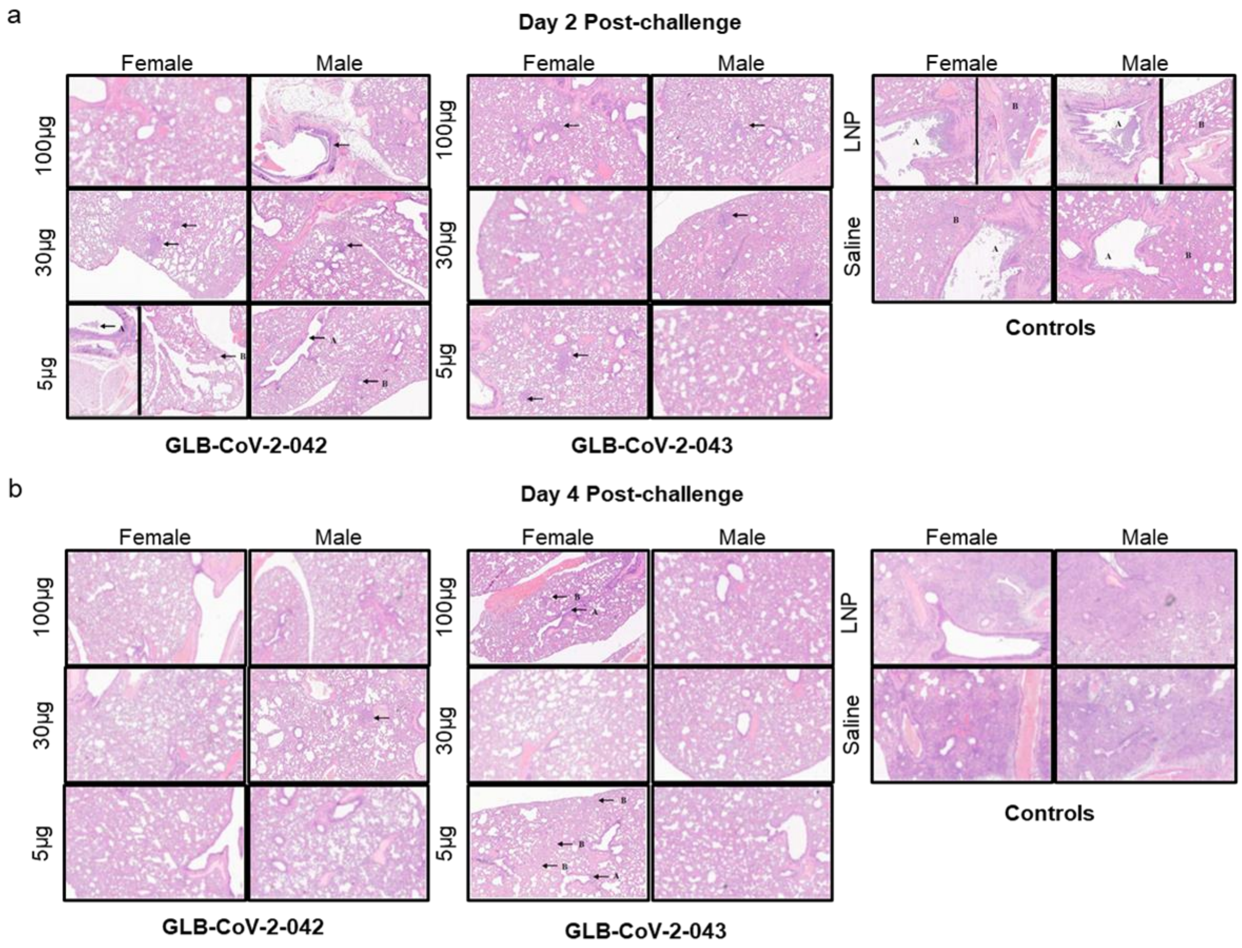

Supplementary Figure 3: Hamster Lung Histopathology following SARS-CoV-2 Challenge

Golden Syrian Hamsters (n=4 male and n=4 female) were vaccinated with 100, 30, or 5 µg of GLB-CoV-2-042 or GLB-CoV-2-043 on days 0 and 21 and challenged on day 42. On days 2 and 4 post-challenge animals were necropsied for tissue collection and terminal bleeds; n=4 per vaccine group per day. (a-b) Histopathological images from GLB-CoV-2 vaccinated and control hamsters of both sexes at days 2 and 4 post-challenge. Arrows indicate necrosis/mixed cell inflammation; A=bronchial/bronchiolar epithelium and B=alveoli.

Day 2 Microscopic Findings																																
	GLB-CoV-2-042												GLB-CoV-043												LNP				Saline			
Dose (µg)	100				30				5				100				30				5				N/A				N/A			
Sex	F	F	M	M	F	F	M	M	F	F	M	M	F	F	M	M	F	F	M	M	F	F	M	M	F	F	M	M	F	F	M	M
Within normal limits	N	N		N		N		N				N				N	N	N		N			N									
Bronchial/bronchiolar epithelium necrosis/mixed cell inflammation										1	1										1			1	2	3	3	3	3	1	3	3
Alveolar necrosis/mixed cell inflammation			2		2		1		1	1	1		1	1	2				1			2			2	3	2	3	3	2	3	3
Alveolar edema																																
Alveolar hemorrhage																																

N = normal; 1 = minimal; 2 = mild; 3 = moderate; 4 = severe; N/A = not applicable

Supplementary Table 1: Hamster Lung Histopathology Scoring Day 2 Post SARS-CoV-2 Challenge

Golden Syrian Hamsters (n=4 male and n=4 female) were vaccinated with 100, 30, or 5 µg of GLB-CoV-2-042 or GLB-CoV-2-043 on days 0 and 21 and challenged on day 42. On day 2 post-challenge animals were necropsied for tissue collection and terminal bleeds; n=4 per vaccine group per day. Histopathology was scored according to severity 45 of tissue damage.

Day 4 Microscopic Findings																																
	GLB-CoV-2-042												GLB-CoV-043												LNP				Saline			
Dose (µg)	100				30				5				100				30				5				N/A				N/A			
Sex	F	F	M	M	F	F	M	M	F	F	M	M	F	F	M	M	F	F	M	M	F	F	M	M	F	F	M	M	F	F	M	M
Within normal limits	N	N		N	N	N		N	N	N	N	N	N		N	N	N	N	N	N	N		N	N								
Bronchial/bronchiolar epithelium necrosis/mixed cell inflammation														1								1			3	3	3	3	3	3	2	3
Alveolar necrosis/mixed cell inflammation							1							1								2			4	4	4	4	4	4	4	4
Alveolar edema																						2			3	2	2	2	2	2	2	2
Alveolar hemorrhage			1																			1			2	3	2	2	2	2	2	2

N = normal; 1 = minimal; 2 = mild; 3 = moderate; 4 = severe; N/A = not applicable

Supplementary Table 2: Hamster Lung Histopathology Scoring Day 4 Post SARS-CoV-2 Challenge Golden Syrian Hamsters (n=4 male and n=4 female) were vaccinated with 100, 30, or 5 µg of GLB-CoV-2-042 or GLB-CoV-2-043 on days 0 and 21 and challenged on day 42. On day 4 post-challenge animals were necropsied for tissue collection and terminal bleeds; n=4 per vaccine group per day. Histopathology was scored according to severity of tissue damage.
